# Wie lassen sich Hürden bei der Versorgung mit digitalen Gesundheitsanwendungen (DiGA) überwinden? Eine Betrachtung des Patientenpfads bei unipolarer Depression

**DOI:** 10.1007/s00103-024-04007-z

**Published:** 2025-02-03

**Authors:** Brit S. Schneider, Florian Koerber, Cordula C. J. Kreuzenbeck, Sophie Brenner

**Affiliations:** IU Internationale Hochschule GmbH, Juri-Gagarin-Ring 152, 99084 Erfurt, Deutschland

**Keywords:** DiGA, Zugang, Adhärenz, Behandlungspfad, Leitlinie, Digital therapeutics, Access, Adherence, Treatment pathway, Guideline

## Abstract

Digitale Gesundheitsanwendungen (DiGA) können eine wichtige Rolle bei der Behandlung von unipolarer Depression spielen. In Deutschland ist der Bedarf hoch, jedoch nutzen weniger als 1 % der Patienten DiGA, was auf bestehende Hürden hinweist. Dieser Artikel gibt einen Überblick über Zugangs- und Nutzungshürden auf dem Patientenpfad und diskutiert Lösungsansätze, um die Verbreitung und Nutzung digitaler Therapien zu verbessern.

Basierend auf der Nationalen VersorgungsLeitlinie (NVL) Unipolare Depression (2022) wurde ein Patientenpfad mit den Phasen „Entscheidung“, „Zugang“ und „Nutzung“ entwickelt. Anhand dieser Struktur wurden auf Basis aktueller Literatur bestehende Versorgungshürden identifiziert sowie Lösungsansätze abgeleitet. Die narrative Übersicht zeigt, dass verschiedene Faktoren den effektiven Einsatz von DiGA beeinträchtigen. In der Entscheidungsphase sind mangelndes Wissen über DiGA und fehlendes Vertrauen in ihre Wirksamkeit zentrale Herausforderungen. In der Zugangsphase stellen Datenschutzbedenken und bürokratische Hürden wesentliche Barrieren dar. Während der Nutzungsphase führen Schwierigkeiten bei der Anwendung und eine geringe Adhärenz zu einer eingeschränkten Wirksamkeit.

Um diese Hürden zu überwinden, werden mehrere Lösungsansätze vorgeschlagen. Eine umfassende Aufklärung über die Wirksamkeit digitaler Therapien könnte das Vertrauen in DiGA stärken. Verbesserungen in der Benutzerfreundlichkeit und eine stärkere Einbindung von Behandelnden könnten die Akzeptanz und Adhärenz erhöhen. Zudem könnten gezielte Schulungen für Fachkräfte und Patienten die Nutzung von DiGA fördern. Diese Erkenntnisse sind auch für andere Anwendungsbereiche digitaler Therapien relevant.

## Abstract (en)

Digital health applications (DiGA) can play an important role in the treatment of unipolar depression. In Germany, the demand is high, but less than 1% of patients use DiGA, indicating existing barriers to their use. This article provides an overview of access and usage barriers along the patient pathway and discusses possible solutions to improve the dissemination and use of digital therapies.

Based on the National Care Guideline (NVL) Unipolar Depression (2022), a patient pathway with the phases of “decision,” “access,” and “usage” was developed. Using this structure, existing care barriers were identified based on current literature, and solutions were derived. The narrative review shows that various factors hinder the effective use of DiGA. In the decision phase, lack of knowledge about DiGA and lack of trust in their effectiveness are central challenges. In the access phase, data protection concerns and bureaucratic hurdles are significant barriers. During the usage phase, difficulties in application and low adherence lead to limited effectiveness.

Several solutions are proposed to overcome these barriers. Comprehensive education about the effectiveness of digital therapies could strengthen trust in DiGA. Improvements in user-friendliness and greater involvement of healthcare providers could increase acceptance and adherence. Additionally, targeted training for professionals and patients could promote the use of DiGA. These insights are also relevant for other areas of digital therapies.

## Einleitung

Unipolare Depression ist eine verbreitete Störung, die durch Interessenverlust, Antriebsminderung und gedrückte Stimmung gekennzeichnet ist. Betroffene haben Schwierigkeiten, alltägliche Aufgaben zu bewältigen, und leiden unter Selbstzweifeln, Konzentrationsproblemen, Grübeln sowie Schlaf- und Appetitstörungen [1]. Depressionen führen oft zu Krankschreibungen und Arbeitsunfähigkeit [2, 3]: Pro Jahr erleben ca. 5,3 Mio. Menschen in Deutschland mindestens eine Episode unipolarer Depression [4, 5].

Eine Herausforderung der psychotherapeutischen Behandlung von Depression sind lange Wartezeiten, besonders in ländlichen Regionen [6]. 2018 betrug die durchschnittliche Wartezeit auf eine Akuttherapie 10,5 Wochen und auf eine Richtlinienpsychotherapie 19,6 Wochen [7], wobei sich je nach methodischer Vorgehensweise und untersuchter Region in der Literatur unterschiedliche Angaben finden [8]. Trotz zuletzt verbesserter Versorgung gibt es insbesondere in ländlichen Gebieten weiterhin Versorgungslücken, die statistisch nicht ausreichend erfasst werden [9]. 2017 wurden nur 8,1 % der Diagnosefälle psychotherapeutisch behandelt, wobei die Behandlungswahrscheinlichkeit mit dem Schweregrad der Depression steigt [7].

Die 2022 überarbeitete Nationale VersorgungsLeitlinie (NVL) für Unipolare Depression in Deutschland empfiehlt nun auch den Einsatz von internet- und mobilbasierten Interventionen (IMI), einschließlich digitaler Gesundheitsanwendungen (DiGA; [1]). DiGA sind gemäß dem Digitale-Versorgung-Gesetz (DVG) Medizinprodukte niedriger Risikoklasse, die auf digitalen Technologien beruhen und der Diagnose, Therapie, Linderung oder Kompensation von Krankheiten, Verletzungen oder Behinderungen dienen. Diese können, nachdem sie im DiGA-Verzeichnis des Bundesinstituts für Arzneimittel und Medizinprodukte (BfArM) gelistet wurden, als Krankenkassenleistung verordnet oder beantragt werden. So sollen die Autonomie und Zufriedenheit der Patienten gestärkt und die Therapieadhärenz erhöht werden. Aufgrund des direkten Zugangs sind DiGA besonders für Patienten mit leichten depressiven Episoden geeignet. DiGA-Angebote, die kognitive Verhaltenstherapie und Achtsamkeitsübungen enthalten, können als niedrigschwellige Angebote den Therapieeinstieg erleichtern. Therapieplätze können so an schwer erkrankte Patienten vergeben werden [1].

Die meisten im DiGA-Verzeichnis gelisteten Anwendungen sind für psychiatrische Indikationen zugelassen (26/55, Stand August 2024), davon 7 gegen Depressionen [10], was auf die hohe Prävalenz und die große Nachfrage nach Psychotherapie zurückzuführen ist. Von September 2020 bis Oktober 2023 wurden 32 % der 374.377 DiGA-Verordnungen für psychische Erkrankungen eingelöst [11]. Bezogen auf eine Zielpopulation von mehreren Millionen Menschen in Deutschland ist die Nutzung von DiGA insgesamt jedoch noch gering [12, 13]: Die beiden etabliertesten Apps deprexis® (GAIA AG, Hamburg) und Selfapy (Online-Therapie bei Depression; Selfapy GmbH, Berlin) wurden bis September 2023 nur etwa ca. 40.000 Mal heruntergeladen [11].

Die klinische Forschung zeigt, dass DiGA Depressionen wirksam behandeln können [14] und depressive Symptome stärker reduzieren als frei verfügbare Apps [15]. Aufgrund der noch niedrigen Nutzerzahlen in Deutschland rücken zunehmend die Akzeptanz und praktische Anwendung von DiGA in den Fokus. Einige neuere Studien ermitteln die Nutzerperspektive für digitale Therapien auf der Grundlage von Stakeholder-Interviews [16–19] oder befassen sich mit Problemen und Hürden von DiGA [20]. Keine dieser Publikationen betrachtet jedoch den gesamten Patientenpfad. Die vorliegende Arbeit gibt einen selektiven Überblick über die in der Literatur diskutierten Hürden, die entlang eines patientenzentrierten und leitlinienbasierten Behandlungspfades auftreten können und die einem verstärkten Einsatz von DiGA in der Versorgung unipolarer Depression entgegenstehen. Anschließend werden für jede der identifizierten Hürden Lösungsansätze für ihre Überwindung diskutiert.

## Methoden

Die Patientenversorgung mit DiGA wird anhand eines Patientenpfads dargestellt. Ein Patientenpfad beschreibt die Versorgungsabläufe für eine spezifische Patientengruppe in einem bestimmten Zeitraum [21]. Hierfür wurden Menschen mit einer erstmaligen leichten akuten Episode einer unipolaren Depression als Patientengruppe gewählt, da die NVL die Nutzung digitaler Therapien für Patienten mit leichtgradiger Depression stark empfiehlt („soll“), für mittelgradige und schwer depressive Patienten jedoch nur als alternativen Therapieansatz [1]. Zudem sind einige DiGA bei schweren depressiven Episoden kontraindiziert [22]. Da der Fokus dieses Artikels auf möglichen Zugangs- und Anwendungshürden von DiGA liegt, werden andere therapeutische Optionen wie Pharmakotherapie nicht weiter berücksichtigt.

Um dem Anspruch eines evidenzbasierten Patientenpfads ausreichend Rechnung zu tragen, wurde die NVL auf allgemeine Therapieempfehlungen untersucht und ein vereinfachter Patientenpfad ohne DiGA abgeleitet. Anschließend wurde dieser Pfad auf Basis der Empfehlungen der NVL zu IMI sowie der in § 33a SGB V genannten Zugangswege um DiGA erweitert und in eine Entscheidungsphase, eine Zugangsphase und eine Nutzungsphase unterteilt. Beide Pfade wurden von einem Therapeuten mit Erfahrung in der Behandlung von Depressionen validiert.

Im Anschluss wurden anhand einschlägiger Literatur iterativ und hypothesengetrieben Herausforderungen, Zugangshürden und Lösungsstrategien recherchiert. Dazu wurden zunächst entlang des Behandlungspfades hypothetische Herausforderungen für die DiGA-Nutzung bei der Therapie von Depression identifiziert. Zu jeder Herausforderung wurde gezielt Literatur gesucht, um ihre Hypothese zu verifizieren und zu vertiefen oder zu falsifizieren. Die Literatur wurde nicht systematisch mit einheitlichen Schlagworten für die gesamte Fragestellung recherchiert, sondern orientiert am Patientenpfad. Diese Vorgehensweise ermöglichte eine Zuordnung der aus der Literatur identifizierten Herausforderungen zu den einzelnen Elementen des Behandlungspfads und eine systematische Darstellung der relevanten Implikationen für die Versorgung. Auf Basis der Ergebnisse wurden schließlich Lösungsansätze und mögliche Ansatzpunkte zur Verbesserung der Versorgung abgeleitet und diskutiert.

Da es bislang nur wenig wissenschaftliche Literatur zu DiGA bei Depression gibt, wurden bei der Recherche auch übertragbare Ergebnisse aus Publikationen zu anderen Indikationen oder frei verfügbaren digitalen Anwendungen für psychische Erkrankungen berücksichtigt. Aufgrund der aktuellen Studienlage geht diese Arbeit davon aus, dass dauerhaft in das DiGA-Verzeichnis aufgenommene DiGA geeignet sind, eine depressive Symptomatik zu verbessern. Voraussetzungen hierfür sind allerdings eine passende Verordnung, eine bewusste Therapieentscheidung, die korrekte Einrichtung der DiGA sowie ein Onboarding des Patienten für eine korrekte Anwendung [17, 23].

## Patientenpfade bei unipolaren Depressionen

### Patientenpfad ohne DiGA-Einsatz.

**Abb. 1:**
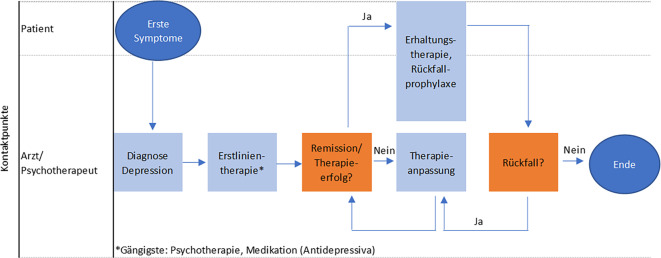
Patientenpfad ohne digitale Gesundheitsanwendungen (*DiGA*) als Therapieoption. (Quelle: eigene Analyse und Abbildung auf Grundlage von [1])

Abb. 1 zeigt zunächst einen stark vereinfachten Behandlungspfad gemäß NVL-Leitlinie ohne DiGA-Einsatz. Dieser beginnt mit dem Auftreten erster Symptome, gefolgt von einem Besuch bei einem Arzt oder Psychotherapeuten (nachfolgend gemeinsam als Behandelnde bezeichnet) zur Diagnosestellung und gemeinsamen Entscheidung über die Erstlinientherapie. Die bislang gängigsten Erstbehandlungen sind Antidepressiva und Psychotherapie, die bei akuten depressiven Symptomen, zur Erhaltungstherapie und zur Vorbeugung von Rückfällen eingesetzt werden können. Weitere Behandlungsoptionen sind Psychoedukation und Training, Neurostimulation, psychosoziale Therapie sowie digitale Therapien (Tab. 1). Der Behandelnde begleitet den Patienten während des gesamten Behandlungsverlaufs, um die Therapie bei Bedarf anpassen zu können.

**Tab. 1:** Therapieoptionen bei unipolarer Depression gemäß Nationaler VersorgungsLeitlinie (*NVL*, 2022). (Quelle: eigene Darstellung auf Grundlage von [1])

A)	Psychoedukation und Schulungen (Bestandteil der Therapieoptionen B–H)
B)^1^	Niedrigintensive Interventionen (z. B. Bibliotherapie, Beratung, Verhaltensaktivierung)
C)^1^	Internet- und mobilbasierte Interventionen (inkl. digitale Gesundheitsanwendungen – DiGA)
D)^1^	Psychotherapie (Verhaltenstherapie, psychodynamische Verfahren, systemische Therapie)
E)	Medikation (Antidepressiva)
F)	Neurostimulatorische Verfahren (konvulsiv, nichtinvasiv transkraniell, invasiv)
G)	Psychosoziale Therapie (z. B. Ergotherapie, Soziotherapie, Selbsthilfe und Peer Support)
H)	Unterstützende nichtmedikamentöse Verfahren (z. B. Lichttherapie, Bewegungstherapie)

^1^ Starke positive Empfehlung („soll“) als Erstlinientherapie für eine erstmalige leichtgradige Episode

### Patientenpfad mit DiGA-Einsatz.

**Abb. 2:**
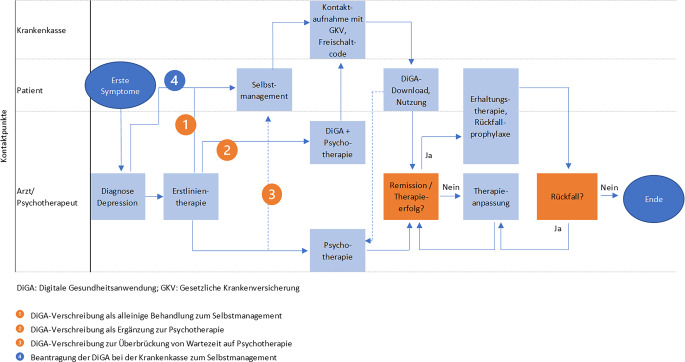
Patientenpfad mit digitalen Gesundheitsanwendungen (*DiGA*) als Therapieoption. (Quelle: eigene Analyse und Abbildung auf Grundlage von [1])

Der Patientenpfad ändert sich grundlegend mit der Einführung einer digitalen Therapie (DiGA) als eigenständige oder ergänzende Therapieoption (Abb. 2). Er beginnt ebenfalls mit den ersten Symptomen und der Depressionsdiagnose, die Voraussetzung für den Zugang zur DiGA ist. Behandelnder und Patient können nun eine der folgenden Optionen für die Nutzung der DiGA wählen:

1. DiGA als alleinige Behandlung zum Selbstmanagement,
2. DiGA als Ergänzung zur Psychotherapie,
3. DiGA zur Überbrückung von Wartezeiten für eine Psychotherapie.

In allen 3 Fällen wird die DiGA durch den Behandelnden verschrieben. Dieser sollte die Therapie begleiten, um die Reaktion des Patienten zu überwachen und die Therapie bei Bedarf anzupassen. Sofern dem Patienten eine entsprechende Diagnose vorliegt, kann dieser die DiGA auch ohne Verordnung durch den Behandelnden bei der Krankenkasse beantragen (Option 4).

In jedem Fall muss der Patient sich anschließend an die Krankenkasse wenden, um den Freischaltcode für die DiGA-Nutzung zu erhalten, die Anwendung herunterladen, installieren und verstehen, wie man sie richtig nutzt.

## Kritische Phasen entlang des Patientenpfades

Die in der Theorie einfache Integration von DiGA in den Behandlungsprozess enthält in der Praxis einige Hürden, die sich entlang des DiGA-Patientenpfades 3 verschiedenen Phasen zuordnen lassen:

- Phase 1: Entscheidungsphase mit Hürden bei der Diagnosestellung und Verordnung (Tab. 2),
- Phase 2: Zugangsphase mit technischen oder organisatorischen Hürden (Tab. 3),
- Phase 3: Nutzungsphase mit Hürden in der Anwendung (Tab. 4).

**Tab. 2:** Herausforderungen in der Entscheidungsphase (Phase 1) bei der Integration digitaler Gesundheitsanwendungen (*DiGA*) in den Behandlungsprozess bei unipolarer Depression. (Quelle: eigene Analyse)

Situation	Hürden	Effekte
Erste Symptome und Suche nach Hilfe	– Angst vor Stigmatisierung, Mangel an Motivation, kulturelle Unterschiede – Fehlendes oder unzureichendes Wissen über DiGA als Therapieoption	Fehlender Zugang zu Therapie (inkl. DiGA)
Diagnosestellung	Risiko einer Fehldiagnose	Kein Zugang zu geeigneter Therapie (inkl. DiGA)
Verschreibung von DiGA: Grundsätzliche Herausforderungen (Nr. 1–3 in Abb. 2)	Fehlendes oder unzureichendes Wissen über DiGA als Therapieoption	Keine Verschreibung, obwohl DiGA indiziert ist und Patient bereit wäre, diese zu nutzen
	Ablehnung der DiGA durch den Behandelnden: – Fehlendes Vertrauen in Wirksamkeit und Qualität der Evidenz – Fehlendes Vertrauen in die eigene Digitalkompetenz – Einarbeitung in Thematik zu aufwendig	
Verschreibung von DiGA als Ergänzung zur Psychotherapie (Nr. 2 in Abb. 2)	Nachfrage nach Psychotherapie übersteigt die Kapazität	Verzögerter Therapiebeginn oder Verwendung von DiGA als alleinige Behandlung, obwohl eine Kombination mit Psychotherapie angezeigt ist
Verschreibung von DiGA zur Überbrückung der Wartezeit (Nr. 3 in Abb. 2)	Fehlendes oder unzureichendes Wissen über DiGA als Therapieoption	Keine Behandlung während der Wartezeit
DiGA als alleinige Behandlung zum Selbstmanagement (Nr. 4 in Abb. 2)	Fehlendes oder unzureichendes Wissen über DiGA als Therapieoption	Kein Zugang zur Therapie

**Tab. 3:** Herausforderungen in der Zugangsphase (Phase 2) bei der Integration digitaler Gesundheitsanwendungen (*DiGA*) in den Behandlungsprozess bei unipolarer Depression. (Quelle: eigene Analyse)

Situation	Hürden	Effekte
Beantragung der DiGA bei der Krankenkasse bzw. Einreichung des Rezepts, Download der DiGA	Beantragung wird als zu aufwendig empfunden; Antriebslosigkeit des Patienten	Verordnung wird nicht eingereicht bzw. keine Beantragung der DiGA
	Mangelnde digitale Kompetenz auf Behandelnden- und Patientenseite	Verordnung wird nicht eingereicht bzw. keine Beantragung der DiGA
	Kein ausreichendes digitales Equipment (Smartphone, Computer) vorhanden	Freischaltcode kann nicht beantragt oder eingelöst werden, Förderung sozialer Ungleichheit
	Informationsdefizit in Bezug auf Komorbiditäten und Kontraindikationen	Antrag abgewiesen, in der Folge Misstrauen gegenüber der Krankenkasse
	Fehlendes Vertrauen in Datenschutz und Datensicherheit	App wird nicht beantragt, heruntergeladen oder genutzt

**Tab. 4:** Herausforderungen in der Nutzungsphase (Phase 3) bei der Integration digitaler Gesundheitsanwendungen (*DiGA*) in den Behandlungsprozess bei unipolarer Depression. (Quelle: eigene Analyse)

Situation	Hürden	Effekte
DiGA-Nutzung	Unzureichende digitale Kompetenz	Misstrauen gegenüber DiGA, Patient verweigert Therapie
	DiGA entspricht nicht den Bedürfnissen des Patienten (Benutzerfreundlichkeit, Design), Patient sucht keine Unterstützung	Patient wendet DiGA nicht ordnungsgemäß an bzw. beendet die Anwendung vor Ablauf der 90 Tage
	Therapeutischer Effekt in der realen Welt unterscheidet sich vom klinischen Studiensetting	DiGA kann die Symptomatik nicht verbessern
Ärztliche/psychotherapeutische Begleitung	Ärztliche/psychotherapeutische Begleitung fehlt oder reicht nicht aus, DiGA-Nutzung nicht leitlinienkonform	Keine Kenntnis über Remission oder Therapieerfolg, mögliche Schäden bei ungeeigneter Behandlung

### Phase 1: Entscheidungsphase

Die erste Phase der Therapieentscheidung ist durch Herausforderungen geprägt, die den Zugang zu einer DiGA erschweren oder verzögern (Tab. 2). Sie beginnt mit behandlungsbedürftigen Symptomen und dem Wunsch nach therapeutischer Unterstützung. Tatsächlich nimmt nur eine von 3 Personen mit Depressionen diese Unterstützung auch in Anspruch [7]. Gründe dafür sind unter anderem Angst vor Stigmatisierung, fehlende persönliche Motivation, kulturelle oder altersbedingte Barrieren [24, 25]. Ohne angemessene Behandlung riskieren die Betroffenen eine Verschlimmerung der Symptome [24, 26] und die Entwicklung von Komorbiditäten [7]. Gerade für Patienten, die keine Psychotherapie in Präsenz wünschen, können DiGA eine niedrigschwellige Therapieoption darstellen. Dieser Schritt erfordert beim Patienten jedoch Kenntnis über DiGA als Therapieoption sowie eine entsprechende Diagnose.

Die zweite Herausforderung in der ersten Phase betrifft die Diagnosestellung und das damit verbundene Risiko einer Fehldiagnose, vor allem in der hausärztlichen Praxis [27]. Dieses Risiko ist bei chronisch kranken Patienten besonders hoch, da durch Komorbiditäten verursachte Symptome die Diagnose erschweren [28]. Tatsächlich leiden bis zu 50 % der chronisch kranken Patienten an behandlungsbedürftigen psychischen Erkrankungen. Häufig wird die Therapie jedoch aufgrund von Fehlinterpretationen der Symptome nicht eingeleitet [7], sodass den Betroffenen der Zugang zu einer geeigneten und leitliniengerechten (DiGA-)Therapie verwehrt bleibt.

Zusätzlich werden in der Literatur einige DiGA-spezifische Hindernisse diskutiert. Hierzu zählt insbesondere mangelndes Wissen der Behandelnden über DiGA als leitliniengerechte Therapieoption. Im Jahr 2022 hatten 14,5 % der ambulant tätigen Behandelnden noch nie von DiGA gehört und 34 % waren nicht bereit, DiGA zu verschreiben [29]. Kreuzenbeck et al. (2024) zufolge stehen 38 % der Behandelnden DiGA insgesamt eher skeptisch gegenüber [13]. Besonders problematisch ist dies bei Hausärzten, die in knapp 60 % der Fälle die Diagnose stellen [28], aber oft unzureichend über DiGA als Therapieoption informiert sind [30]. Nur die Hälfte der Hausärzte kennt die NVL [28] und nicht alle Behandelnden wissen, unter welchen Bedingungen DiGA bewilligt werden [31]. Die Einarbeitung in neue Therapieprogramme gilt als aufwendig und zeitintensiv [32, 33]. Dies birgt das Risiko, dass DiGA trotz Depressionsdiagnose und NVL-Empfehlung nicht verordnet werden.

Ein weiteres Hindernis ist die als unzureichend wahrgenommene klinische Evidenz, die sich negativ auf die Akzeptanz der DiGA auswirkt [16, 31]. In einer Befragung aus dem Jahr 2022 schätzten nur 37,1 % der Behandelnden DiGA als wirksam für die Behandlung von Depressionen ein, 6 % lehnten DiGA aufgrund fehlender klinischer Evidenz grundsätzlich ab [29]. Hinzu kommt das Risiko, dass Behandelnde von einer Verordnung absehen, wenn sie ihre eigene Digitalkompetenz als eher gering einstufen. So nennen 26,8 % der ambulant tätigen Ärzte und Psychotherapeuten technische Hürden bei der Rezepterstellung als großes Hemmnis für den Einsatz von DiGA [29].

Zwar sind DiGA meist für die eigenständige Anwendung durch die Patienten konzipiert und können bei entsprechender Diagnose als eigenständige Behandlungsoption ohne ärztliche oder psychotherapeutische Begleitung genutzt werden (Nr. 1 und 4 in Abb. 2), für die Beantragung ohne Rezept (Nr. 4 in Abb. 2) ist aber die Eigeninitiative der Patienten erforderlich. Sollte der Behandelnde DiGA ablehnen, könnte sich dies aufgrund der Symptomatik depressiver Patienten als unüberwindbare Hürde erweisen.

Gemäß NVL kann die DiGA auch begleitend zur Psychotherapie (Nr. 2 in Abb. 2) eingesetzt werden [1]. Einige DiGA können zudem explizit zur Überbrückung von Wartezeiten (Nr. 3 in Abb. 2) genutzt werden [34], was insbesondere für Patienten aus unterversorgten ländlichen Regionen hilfreich ist. Voraussetzung hierfür ist, dass Behandelnde ausreichend über die Einsatzmöglichkeiten von DiGA informiert sind. Da die Wahrscheinlichkeit einer DiGA-Verordnung jedoch mit zunehmender Bevölkerungsdichte steigt [12], besteht das Risiko, dass Patienten in ländlichen Regionen mit langen Wartezeiten weniger von der Digitalisierung profitieren. Zudem könnten DiGA in unterversorgten Regionen als Ersatz für Psychotherapie eingesetzt werden, obwohl eine begleitende Nutzung zielführender wäre.

### Phase 2: Zugangsphase

Die zweite Phase betrifft technische und organisatorische Hürden nach der Therapieentscheidung (Tab. 3). Unabhängig vom gewählten Behandlungspfad muss der Patient selbstständig an seine Krankenkasse herantreten und das Rezept für die DiGA elektronisch oder postalisch einreichen (Nr. 1–3 in Abb. 2) oder diese direkt beantragen (Nr. 4 in Abb. 2). Hierfür ist eine gültige medizinische Diagnose sowie je nach DiGA ein Beleg für das Fehlen von Kontraindikationen erforderlich (§ 33a SGB V). Depressive Patienten dürften jedoch Schwierigkeiten haben, sich eigenverantwortlich um die Auswahl einer geeigneten DiGA, die Beantragung sowie die Beschaffung der erforderlichen Nachweise zu kümmern. Aber auch im Falle einer ärztlichen Verordnung besteht die Gefahr, dass es nicht zu einer tatsächlichen Nutzung der DiGA kommt. Einer Umfrage zufolge zweifeln 50 % der ambulant tätigen Ärzte und Psychotherapeuten an der Motivation der Patienten, die Freischaltcodes einzulösen [29]. Über alle DiGA hinweg werden schätzungsweise 8–19 % der genehmigten Freischaltcodes nicht genutzt [11, 35]. Das Phänomen der primären Non-Adhärenz ist allerdings nicht DiGA-spezifisch: Schätzungsweise 20 % der Rezepte für Medikamente werden ebenfalls nicht eingelöst [36, 37].

Eine weitere Hürde betrifft eine mangelnde Digitalkompetenz sowie eine unzureichende technische Ausstattung (Smartphone, Computer, stabile Internetverbindung, Mindestanforderungen an Betriebssysteme) auf Patientenseite. Insbesondere ältere Menschen sind hiervon betroffen – 2020 nutzten beispielsweise nur 52 % der über 70-Jährigen ein Smartphone [33]. Eine technische Überforderung der Patienten könnte dazu führen, dass diese die Verordnung gar nicht erst einreichen. Diese Kluft im Bereich der digitalen Gesundheit (Digital Divide) führt dazu, dass digitaler Inklusion zukünftig eine wachsende Bedeutung zukommt [38].

Probleme beim Zugang zu DiGA können auch durch Komorbiditäten entstehen, bei denen DiGA kontraindiziert sind und die ohne Nutzung der elektronischen Patientenakte oft unbekannt bleiben. Die meisten DiGA sind für die Behandlung leichter bis moderater depressiver Episoden zugelassen und schließen andere, häufig mit Depressionen einhergehende psychische Erkrankungen [39] explizit aus [22]. Zudem scheint den verordnenden Behandelnden nicht immer bewusst zu sein, dass Diagnosen und Kontraindikationen mit der Verordnung dokumentiert werden müssen [12], da es sonst zu einer Ablehnung durch die Krankenkasse kommen kann. Misstrauen gegenüber der Krankenkasse könnte so erzeugt, der Behandlungsbeginn verzögert oder der Krankheitsverlauf verschlimmert werden.

Bereits anderenorts diskutiert ist das fehlende Vertrauen in Datenschutz und Datensicherheit bei digitalen Lösungen [20]. Bis 2024 reichte eine Herstellerselbstauskunft als Nachweis für den Datenschutz einer DiGA. Einzelne Datenschutzverletzungen in der Vergangenheit führten diesbezüglich jedoch zu verstärktem Misstrauen [31]. Um diesem entgegenzuwirken, ist seit August 2024 gemäß § 139e SGB V ein mit der Datenschutz-Grundverordnung (DSGVO) konformes Datenschutzzertifikat erforderlich. Ab 2025 muss zudem die Datensicherheit zertifiziert sein.

### Phase 3: Nutzungsphase

Die Herausforderungen in Phase 3 beziehen sich auf den psychotherapeutischen Einsatz von DiGA durch die Patienten und die Therapiebegleitung durch die Behandelnden (Tab. 4).

Der Nutzen digitaler Therapien wird von der Mehrheit der Behandelnden in der gestärkten Eigenverantwortung der Patienten gesehen [40]. Allerdings sind Apps nicht immer intuitiv und benutzerfreundlich gestaltet. So finden 40 % der Behandelnden, dass DiGA zu kompliziert für Patienten sind [13]. Förderlich für die Nutzung wirken personalisierte Inhalte, Feedbackfunktionen, Features zur Motivation, positive Verstärkung wünschenswerter Gedanken oder Aktivitäten, Gamifizierung mit Anreizen oder Belohnungssystemen sowie die Möglichkeit zu Kommunikation und Austausch innerhalb der App – beispielsweise mit Peer-Gruppen, dem Behandelnden oder über immer erreichbare, intuitive Chatbots [41–44]. Ungeeignete oder fehleranfällige DiGA erhöhen hingegen das Risiko eines Therapieabbruchs und somit negativer gesundheitlicher Folgen.

Damit DiGA überhaupt richtig genutzt werden können, sind digitale Kompetenzen notwendig. Funktionsprobleme können sonst zu einem Therapieabbruch führen, obwohl diese leicht zu beheben wären. Insbesondere für ältere Patienten können digitale Tools daher eine zusätzliche Belastung darstellen und Stress verursachen, vor allem in akuten oder kritischen Situationen [17]. Daher profitieren aktuell hauptsächlich technikaffine Patienten sowie jüngere Menschen von der Digitalisierung [11, 45], was sich auch in den Verordnungszahlen widerspiegelt. Während das Durchschnittsalter der Zielpopulation einer DiGA gegen Depressionen bei 54,0 Jahren liegt, beträgt das Durchschnittsalter der Nutzer nach Verordnung lediglich 42,1 (deprexis®) bzw. 40,0 Jahre (Selfapy; [12]).

Diese Aspekte können zu unsachgemäßer Nutzung oder vorzeitigem Abbruch der Therapie führen, wodurch sich der therapeutische Effekt im Alltag möglicherweise vom klinischen Studiensetting unterscheidet. Studien zeigen, dass die Therapieadhärenz insbesondere bei unbegleiteten Apps gegen psychische Erkrankungen gering ist [23]. So beenden 28,3 % der Nutzer von DiGA gegen Depression die Nutzung entgegen der Empfehlung von mindestens 90 Tagen bereits vor Ablauf eines Monats [12] und auch die Nutzungsdaten frei verfügbarer Apps gegen psychische Erkrankungen zeigen bereits nach wenigen Tagen einen starken Abfall der täglichen Nutzungsrate [44]. Vor allem bei fehlenden Ergebnissen, einer Verschlechterung des Gesundheitszustands und bei besonders jungen oder älteren Nutzern steigt das Risiko eines vorzeitigen Abbruchs [17, 46, 47], wobei auch 18 % der Patienten eine kognitive Verhaltenstherapie (KVT) in Präsenz abbrechen [48].

Ein möglicher Grund für Therapieabbrüche ist die fehlende Einbindung der Behandelnden, da DiGA ausschließlich für die Nutzung durch die Patienten gedacht sind [16, 34]. Die Nutzung ohne ärztliche oder psychotherapeutische Anleitung kann jedoch zu einer nichtleitliniengerechten Behandlung und suboptimaler Therapieadhärenz führen [49]. So zeigen psychotherapeutische DiGA sowie andere digitale Therapien gegen Depression mit therapeutischer Begleitung in Wirksamkeitsstudien bessere Ergebnisse [14, 16, 32] und eine intensivere Nutzung [44]. Daher rät die NVL zur Patientenaufklärung und Verordnung von DiGA durch Ärzte und Psychotherapeuten [1], um das Risiko von Über‑, Unter- und Fehlversorgung sowie möglicher Schäden bei falscher Nutzung zu minimieren.

## Zentrale Herausforderungen für die Nutzung von DiGA

In dieser Arbeit wurden verschiedene Studien zu den Hürden der DiGA-Nutzung synthetisiert und erstmalig anhand eines patientenzentrierten, leitlinienbasierten Behandlungspfades systematisiert. Diese Vorgehensweise erlaubt einen ganzheitlichen Blick auf bestehende Hürden und ermöglicht die Ableitung effektiver Lösungsansätze.

DiGA verändern den Behandlungspfad für Depressionen im Vergleich zur traditionellen Therapie, vor allem, wenn diese als eigenständige Behandlung eingesetzt werden. Im Rahmen der Patientenpfadanalyse wurden die folgenden Kernprobleme entlang der 3 untersuchten Phasen (Entscheidung, Zugang, Nutzung) identifiziert:

1. Informationsdefizite: Unzureichende Kenntnis über DiGA als Therapieoption bei Patienten und Behandelnden führt zu verpassten Chancen für eine angemessene Nutzung. Dies ist oft auf eine ungenügende Bewerbung der Apps durch Behandelnde und Krankenversicherungen zurückzuführen.
2. Zweifel an der Wirksamkeit: Aufgrund des im Vergleich zu Arzneimitteln flexibleren Zulassungsverfahrens für DiGA ist die Evidenz für ihre medizinische Wirksamkeit uneinheitlich. Insbesondere bei vorläufig zugelassenen DiGA kann das Vertrauen in ihre Wirksamkeit fehlen. Zudem wird eine Übertragbarkeit der Studienergebnisse auf die Anwendung in realen Behandlungssettings angezweifelt.
3. Mangelndes Vertrauen in digitale Therapien: Bei Zweifeln an der Wirksamkeit fehlt das Vertrauen in den Therapieerfolg. Viele Behandelnde glauben zudem nicht an eine ausreichende Adhärenz der Patienten.
4. Unzureichende digitale Kompetenz: Patienten und Behandelnde benötigen digitale Kompetenzen für den effektiven Einsatz von DiGA. Insbesondere ältere Menschen werden oft nicht ausreichend ermutigt und befähigt, weshalb es zunehmend zu einer Kluft zwischen den Generationen kommt.
5. Unzureichende Alltagskompetenz bei Depression: Die Diagnose Depression kann es erschweren, eine DiGA zu wählen, einzurichten und effektiv zu nutzen. Die Nutzung selbst kann Stress verursachen oder enttäuschen, was zur Verschlimmerung des Krankheitsbildes und zu Therapieabbruch führen kann.

## Lösungsansätze zur Erhöhung der Nutzung von DiGA

Die Ergebnisse der Analyse zur Evidenz über die Nutzung von DiGA unterstreichen die Notwendigkeit einer intensiveren Kommunikation und Information rund um DiGA. Bislang dient hauptsächlich das DiGA-Verzeichnis als Informationsquelle [31]. DiGA als Medizinprodukte niedriger Risikoklasse dürfen jedoch grundsätzlich auch gegenüber Laien beworben werden, weshalb Hersteller verstärkt zielgruppenspezifische Kommunikationsmaßnahmen einsetzen sollten. Ärzte und Psychotherapeuten spielen eine Schlüsselrolle bei der Patientenaufklärung, sind aber selbst häufig noch unzureichend über DiGA informiert [47]. Im Kontext hoher Arbeitsbelastung, geringer Affinität für digitale Tools und einem aus Sicht der Behandelnden teilweise fehlenden Vertrauen in die Effektivität von DiGA wäre es sinnvoll, etablierte Kommunikationswege wie kassenärztliche Vereinigungen, Fachverbände und Kongresse stärker zu nutzen und dabei auf die bestehenden Standards der Evidenzqualität bei dauerhaft zugelassenen DiGA hinzuweisen. Für die Zukunft wird zwar erwartet, dass sich die teils noch restriktive Haltung von Ärzten und Psychotherapeuten aufgrund von demografischen Veränderungen abschwächen könnte [32]. Dieser Effekt ließe sich durch eine zielgerichtete Kommunikationsstrategie jedoch beschleunigen.

Weiterhin sollte die Rolle des Behandelnden in der digitalen Therapiebegleitung gestärkt werden. Obwohl DiGA eigenständige digitale Therapeutika sind, benötigen sie Erklärung und Korrektur durch den Behandelnden. Diese Einbindung ist entscheidend für den Therapieerfolg und die Adhärenz. Entsprechende Blended-Therapy-Ansätze befinden sich in Deutschland derzeit in der Entwicklung [50]. Behandelnde benötigen zudem einen direkten Zugang zu wichtigen DiGA-Verordnungsinformationen und einen schnellen Zugriff auf Patienteninformationen. Zur Akzeptanzgewinnung muss stärker auf Bedenken von Patienten und Behandelnden eingegangen werden.

Die oft geringe Digitalkompetenz von älteren Patienten und die mentale Belastung von Menschen mit Depression erfordern, dass DiGA eine gute Nutzererfahrung bieten. Für eine hohe Adhärenz und niedrige Abbruchraten muss die digitale Therapie benutzerfreundlich sein und neben evidenzbasierter Verhaltenstherapie entsprechende Designstrategien, z. B. in Form von personalisierten Inhalten oder motivierenden Features, berücksichtigen.

Die wirksamste Nutzung von DiGA liegt dort, wo der Bedarf und die Erfolgschancen am höchsten sind. In ländlichen Gebieten mit langen Wartezeiten und Anfahrtswegen könnten kassenärztliche Vereinigungen oder Verbände die Nutzung von DiGA fördern, um Versorgungslücken zu füllen, ohne Überversorgung oder Verdrängungswettbewerb zu riskieren. Bestimmte Patientengruppen ziehen die räumliche und zeitliche Flexibilität sowie Anonymität einer DiGA einer persönlichen Therapie vor. Eine gezielte Versorgung dieser Patienten mit einer passenden DiGA kann die Zufriedenheit und den Therapieerfolg steigern. Gleichzeitig kann die knappe psychotherapeutische Kapazität gezielt für Patienten eingesetzt werden, die am meisten von ihr profitieren.

## Fazit und Ausblick

Unsere Recherche zeigt für Patienten und Behandelnde mehrere Hürden für die DiGA-Therapie bei unipolarer Depression: fehlendes Bewusstsein für DiGA, Zweifel an der Wirksamkeit, mangelndes Vertrauen in eine digitale Therapie, unzureichende Kompetenz im Umgang mit digitalen Tools und Hürden in der Bewältigung des Alltags.

DiGA sind ein Instrument in der therapeutischen „Toolbox“ von Ärzten und Psychotherapeuten. Für mehr Akzeptanz und eine breitere Nutzung von DiGA müssen Patienten und Behandelnde besser über die Anwendung und Vorteile von DiGA informiert werden. Interessensverbände können dabei als verlässliche Informationsquellen dienen. Die Rolle des Behandelnden in der digitalen Therapie sollte gestärkt werden, um DiGA besser in die Versorgung zu integrieren. Zudem sollten DiGA eine gute Nutzererfahrung bieten, um Akzeptanz und Adhärenz der Patienten mit Depression zu fördern.

Aus der Analyse wird deutlich, dass eine Zulassung von DiGA als Krankenkassenleistung nicht ausreicht, um einen positiven Versorgungseffekt zu erzielen. Vielmehr müssen alle Beteiligten – Patienten und Behandelnde – noch besser über die Vorteile von DiGA informiert werden. Darüber hinaus sind weitere Studien erforderlich, um die Wirksamkeit von DiGA in realen Lebenswelten zu erforschen und eine Übertragbarkeit der Erkenntnisse auf andere Indikationen zu prüfen. Bislang wird in den identifizierten Quellen zudem hauptsächlich die Perspektive der Behandelnden berücksichtigt, während die Nutzerperspektive noch nicht ausreichend untersucht ist. Weitere Forschung ist daher nötig, um detailliertere Erkenntnisse über die Eignung und Passgenauigkeit von DiGA für die Behandlung von Depression zu gewinnen.
